# Evaluation of the morphometric covariation between palatal and craniofacial skeletal morphology in class III malocclusion growing subjects

**DOI:** 10.1186/s12903-020-01140-4

**Published:** 2020-05-27

**Authors:** V. Paoloni, G. Gastaldi, L. Franchi, F. C. De razza, P. Cozza

**Affiliations:** 1 Department of Clinical Sciences and Translational Medicine, University of Rome Tor Vergata, Rome, Italy; 2 Department of Orthodontics, University Vita-Salute San Raffaele, Milan, Italy; 3 Department of Surgery and Translational Medicine, University of Florence, Florence, Italy

**Keywords:** Class III malocclusion, Skeletal 3D covariation, Growing subjects, Geometric morphometric analysis

## Abstract

**Background:**

To study the covariation between palatal and craniofacial skeletal morphology in Class III growing patients through geometric morphometric analysis (GMM).

**Methods:**

In this retrospective study, 54 Class III subjects (24F,30M;7.6 ± 0.8yy) were enrolled following these inclusion criteria: European ancestry, Class III skeletal and dental relationship, early mixed dentition, prepubertal skeletal maturation, familiarity for Class III malocclusion, no pseudo Class III malocclusion. Each patient provided upper digital cast and cephalogram before starting the therapy. Landmarks and semilandmarks were digitized (239 on the casts;121 on the lateral radiographs) and GMM was used. Procrustes analysis and principal component analysis (PCA) were applied to show the principal components of palatal and craniofacial skeletal shape variation. Two-block partial least squares analysis (PLS) was used to assess pattern of covariation between palatal and craniofacial morphology.

**Results:**

Regarding palatal shape variation, PC with largest variance (PC1) described morphological changes in the three space dimensions, while, concerning the craniofacial complex components, PC1 revealed morphological differences along the vertical plane. A significant covariation was found between palatal and craniofacial shape. PLS1 accounted for more than 61,7% of the whole covariation, correlating the craniofacial divergence to palatal height and width.

**Conclusions:**

In Class III subjects increments of angle divergence are related to a narrow and high palate.

## Background

Class III malocclusion is a manifestation of both environmental and genetic interaction on the development of the craniofacial complex [1–5].

Broad diversity exists in the size and shape of the dental and skeletal components contributing to Class III malocclusion. In the literature numerous authors [6–10] studied this topic in order to allow a correct orthodontic diagnosis and treatment planning in Class III patients.

Lateral cephalogram has been the standard record used in studies attempting to quantify the underlying craniofacial morphology in Class III subjects. Guyer et al. showed that less than 20% of Class III malocclusion had pure mandibular prognathism while 25% had pure maxillary skeletal retrognathism, and 22% had a combination of the two [6]. Ellis and McNamara noted that the most common combination of variables (30.1%) was a protrusive mandible, retrusive maxilla, long lower face height, protrusive maxillary incisors and retrusive mandibular incisors [7]. Class III subjects presented also a more obtuse gonial angle, a forward position of the glenoid fossa and a short anterior cranial base [8, 9].

Chen et al. [10] analysed the development of the dental arches and the skeletal mandibular- maxillary bases in untreated subjects with Class III malocclusions. They found that the maxillary skeletal base widths and the maxillary intermolar widths were significantly smaller in the Class III group than in the control Class I group, while the mandibular intermolar widths showed no significant differences between the examined groups.

However, the conventional cephalometry, based on angular and linear measurements, and the conventional dental casts analysis have proved to be insufficient for the analysis of shape changes of complex anatomical forms [11]. Moreover, palatal morphology is associated with the different aspects of maxillofacial morphology.

In recent times geometric morphometric analysis (GMM) has become more important in orthodontics as a means of investigating modifications in skeletal and dental morphology that can explain complex shape differences more successfully than coefficients from traditional morphometric analysis [12, 13].

GMM was used by Parcha et al. [14] in order to analyse the covariation between palatal morphology and craniofacial skeletal pattern in a general orthodontic population, while by Paoloni et al. [15] in Class II growing patients.

Ahn et al. [16] analysed the relationship between the morphology of the palate and the facial skeletal patterns using the structural equation modelling (SEM) in adults with Class III malocclusion.

To our knowledge no data are available with regard to the 3D evaluation of the relationships between the morphology of the palate and the facial skeletal patterns in growing subjects with Class III malocclusion.

Therefore, the aim of the present retrospective research was to analyse the patterns of covariation between palatal and craniofacial morphology in Class III growing patients, in early mixed dentition, assessed by the tools of geometric morphometric analysis (GMM).

## Methods

Fifty-four class III subjects (24 females and 30 males; mean age 7.6 ± 0.8 years) were enrolled retrospectively from the departments of orthodontics of the universities of Rome Tor Vergata and Florence

The inclusion criteria were: European ancestry (white), Class III skeletal relationship (ANB < 0°, Wits appraisal < − 2 mm), Class III molar relationship, early mixed dentition, prepubertal skeletal maturation (CS1 - CS2) [17], familiarity for Class III malocclusion, presence of pretreatment records (digital casts and lateral radiographs). Time limit between digital casts and lateral radiographs was set within 6 months.

Exclusion criteria were: anterior functional shift (pseudo Class III malocclusion), unilateral crossbite with lateral functional mandibular shift, pubertal and postpubertal subjects (older than CS3), deciduous and permanent dentition, previous orthodontic treatment, sucking habits or mouth breathing, multiple and/or advanced caries, tooth anomalies, cleft lip and/or palate, genetic diseases.

The study was authorized by the Ethical Committee of the University of Rome Tor Vergata (Protocol number: 201/19) and patients’ parents gave their informed written consent.

Upper study casts and lateral cephalograms were required before any treatment for every patient.

In order to analyse the palatal morphology, study casts of all subjects were scanned using an extraoral scanner OrthoXscan (OrthoXscan; Dentaurum GmbH and co, Ispringen, Germany) with a manufacturer’s reported accuracy of 20 μm. All models were exported in a Standard Tesselation Language format (.stl digital file). Lateral radiographs were digitally collected with a resolution of 150 dpi and converted to real size. 3D GMM was used [18–20] to evaluate the palatal and the craniofacial skeletal morphology. Pretreatment digital casts and cephalograms were digitized through Viewbox 4 software (dHAL software, Kifissia, Greece).

**Fig. 1:**
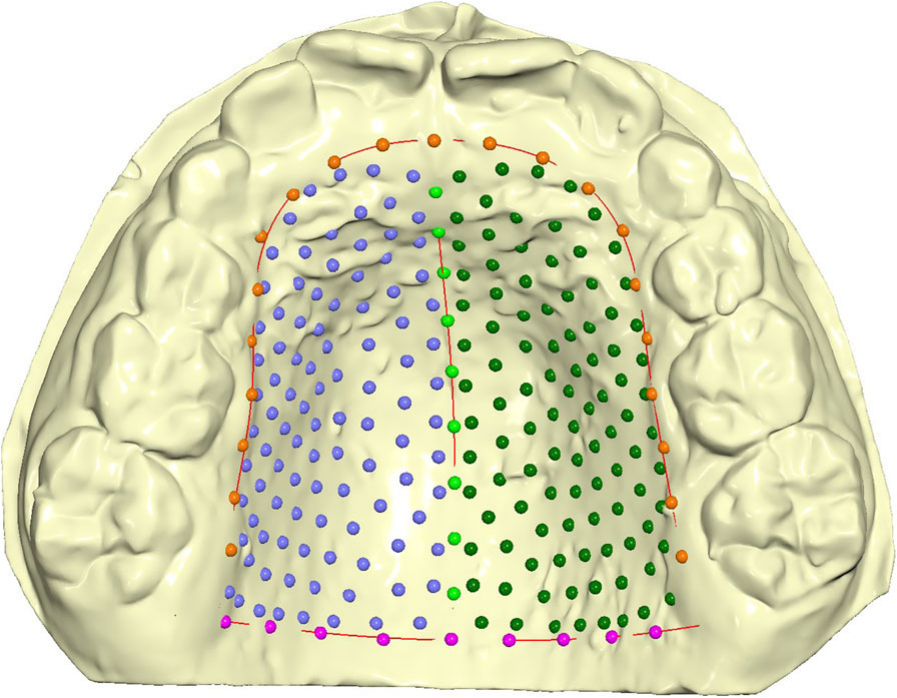
The three curves drawn on the digital casts. Green points: midsagittal suture; orange: perimeter of the dental arch on margin; pink: posterior border tangent to the distal surface of permanent first molars; dark green and blue: semilandmarks on the palatal surface

The digital cast dataset was made of three curves and a total of 239 landmarks (Fig. 1). The palatal boundaries were assesed as: the midsagittal suture (9 points), the perimeter curve of the upper arch passing apical to the gingival sulci of each tooth (21 points) and the posterior curve passing from distal of the first permanent molars, perpendicular to the midsagittal line (9 points). The other points (semilandmarks) were positioned uniformly on the maxillary surface within the confines delimited by the three curves [12, 14, 15].

**Fig. 2:**
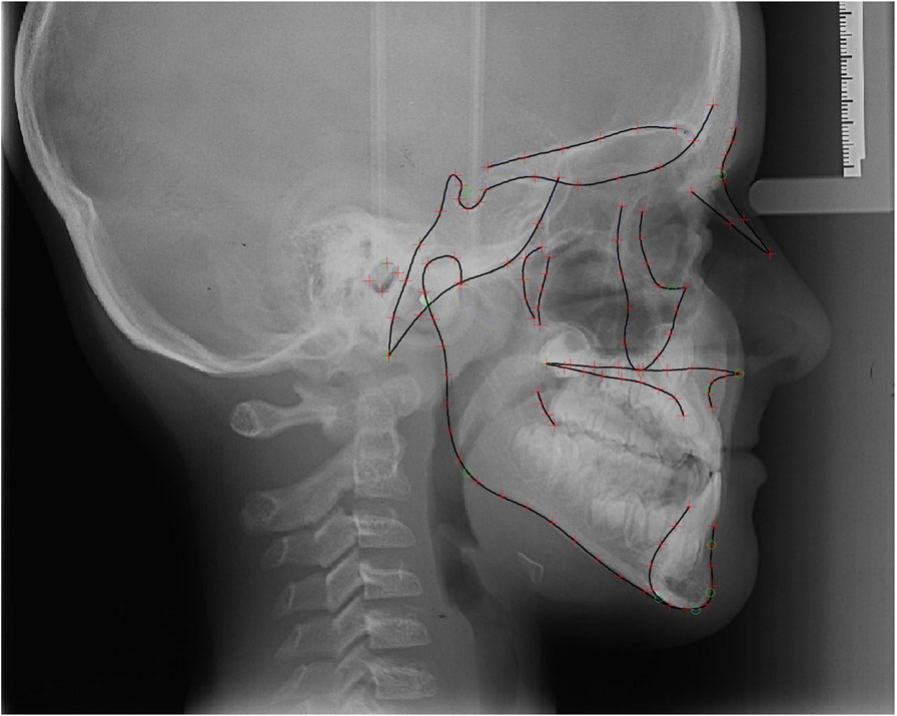
Fixed landmarks (green circles) and sliding semilandmarks (red crosses) used to describe the craniofacial skeletal complex

The craniofacial skeletal shape was analysed through 15 continuous curves and 121 points (14 fixed cephalometric landmarks, 107 semilandmarks positioned at equidistant distances along the curves) [14, 15] (Fig. 2).

Palatal and craniofacial datasets averages were measured and used as a fixed reference (Procrustes average) to allow all semilandmarks to slide and become more homologous from patient to patient in order to reduce the thin-plate spline (TPS) bending energy [21, 22]. This sliding was repeated two times.

All digitizations of cephalograms and digital casts were done by the same operator (D.R.F.C) and analysed through the Generalized Procrustes means.

### Statistical analysis

To evaluate the method reproducibility, 20 upper casts and 20 lateral radiographs were randomly selected and re-digitized by the same operator 10 days after the first digitization. Random error was expressed as the distance between repeated digitizations in shape space compared with the total variance of the sample [15]. Generalized partial least square Procrustes superimposition was performed to extract Procrustes coordinates for shape description and principal component analysis (PCA) was applied to reveal the main patterns of maxillary and of craniofacial skeletal morphologic variation. Two-block partial least squares analysis (PLS) was used to evaluate any shape covariation between palatal and craniofacial morphology. The evaluation was done using Viewbox 4 (PCA) and MorphoJ software (PLS) and covariation strength was assesed by the RV coefficient of Escoufier [23] (10,000 permutations) as a scalar measure of the strength of association between the coordinates of two sets of landmarks [24].

## Results

Mean random error of the 20 repeated digitizations was 6.4% for upper digital casts and 8.7% for lateral radiographs.

As for the evaluation of the palatal shape, the first 3 principal components (PCs) were considered to be statistically meaningful (at least 5% of total shape variability) and explained the 69,6% of the total shape variability.

**Fig. 3:**
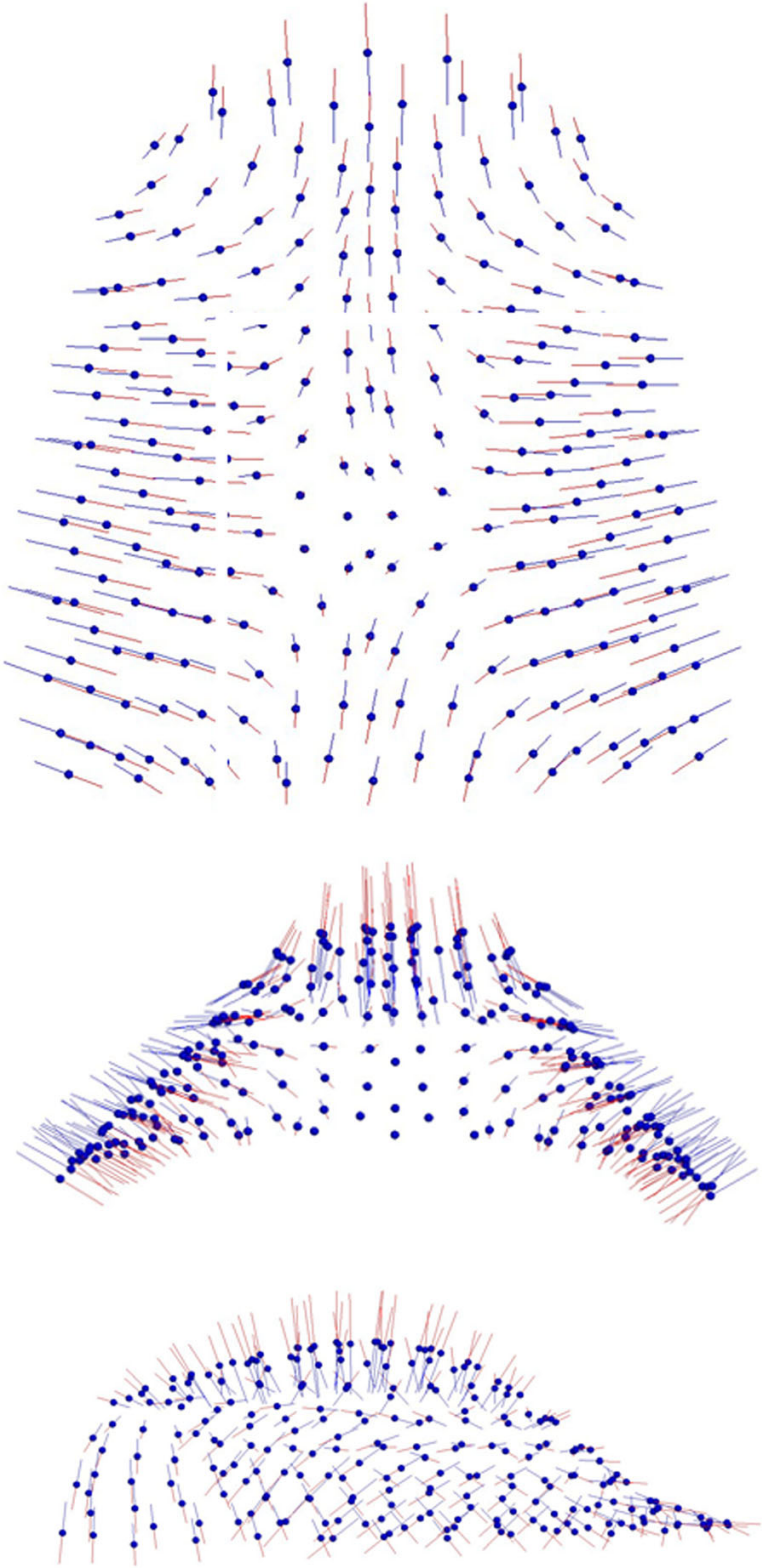
Graphic depiction of the PC1 of the palate from the three views: the main shape differences consisted of larger and shorter palatal vault height vs narrower and higher palate

**Fig. 4:**
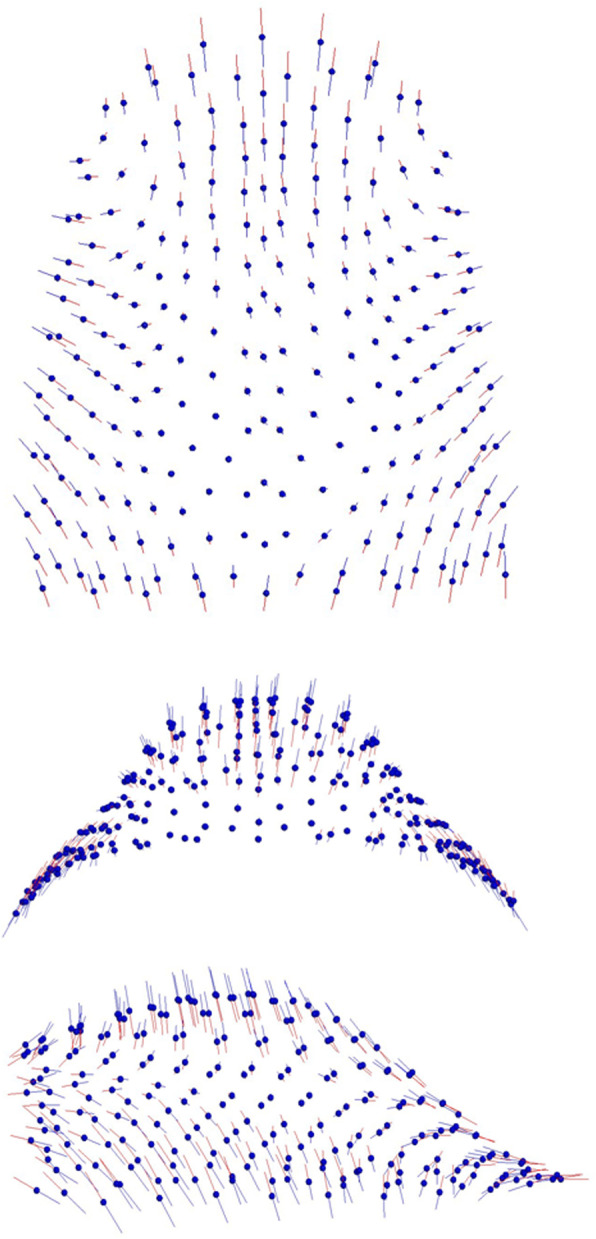
Graphic depiction of the PC2 of the palate from the three views: shape differences were related to the palatal height

PC1 showed the largest variance (43,5%) and described morphological changes in all the three dimensions of the space, especially along the vertical and transversal planes. As shown in Fig. 3, palatal variation pattern associated high palates to narrow ones, while wide palates looked shallow. PC2 (17,6%) expressed significant shape differences only in palatal height (Fig. 4).

As for the craniofacial components morphology, the first 4 PCs were statistically meaningful and showed the 60,5% of the total shape variability.

**Fig. 5:**
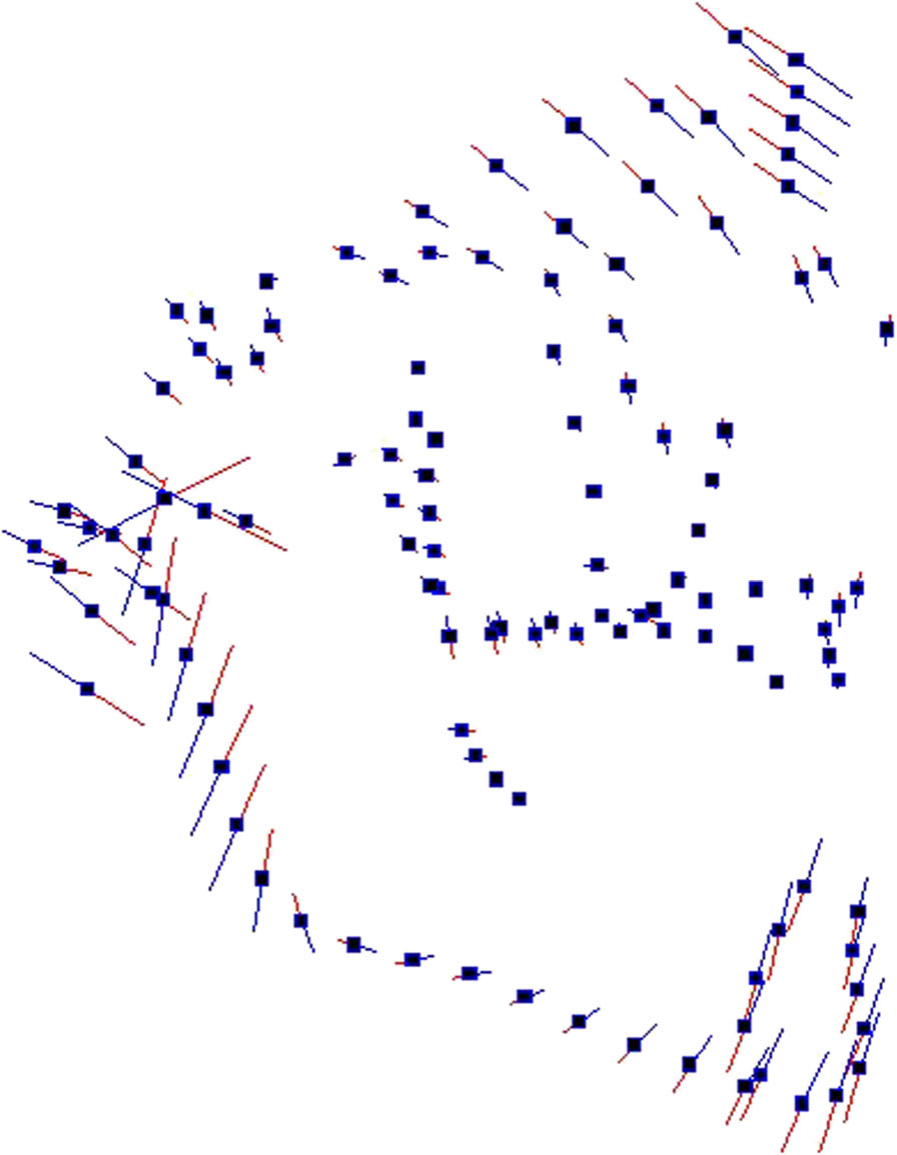
Graphic depiction of the PC1 of the craniofacial complex: the main shape differences were mainly in the vertical plane

**Fig. 6:**
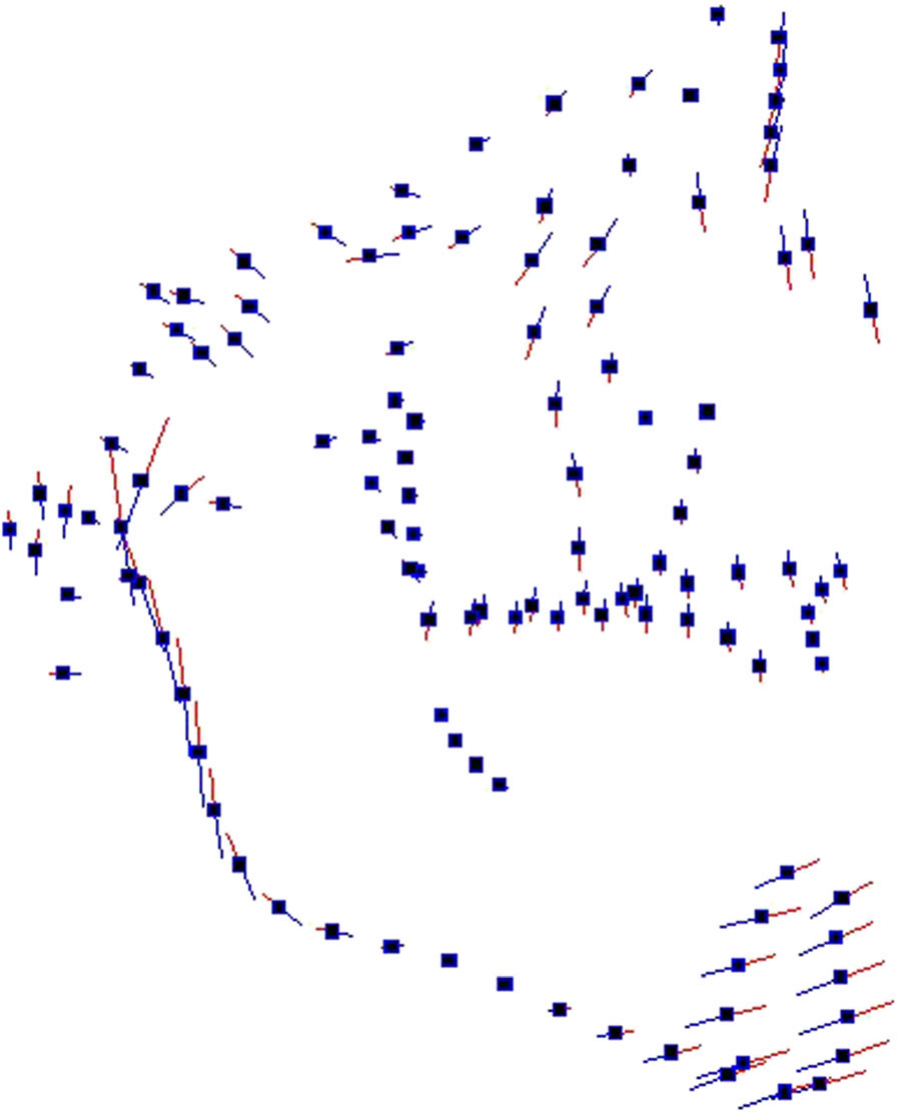
Graphic depiction of the PC2 of the craniofacial complex: shape differences were mainly in the sagittal plane

PC1 (25,8%) showed shape differences on the vertical plane, especially located in the mandibular ramus and in the condylar and symphyseal mandibular regions (Fig. 5). PC2 (15,5%) referred to the sagittal plane with shape differences more evident in the mandibula in relation to the cranial base (Fig. 6).

PLS analysis assessed the covariance between palatal and craniofacial skeletal components.

**Fig. 7:**
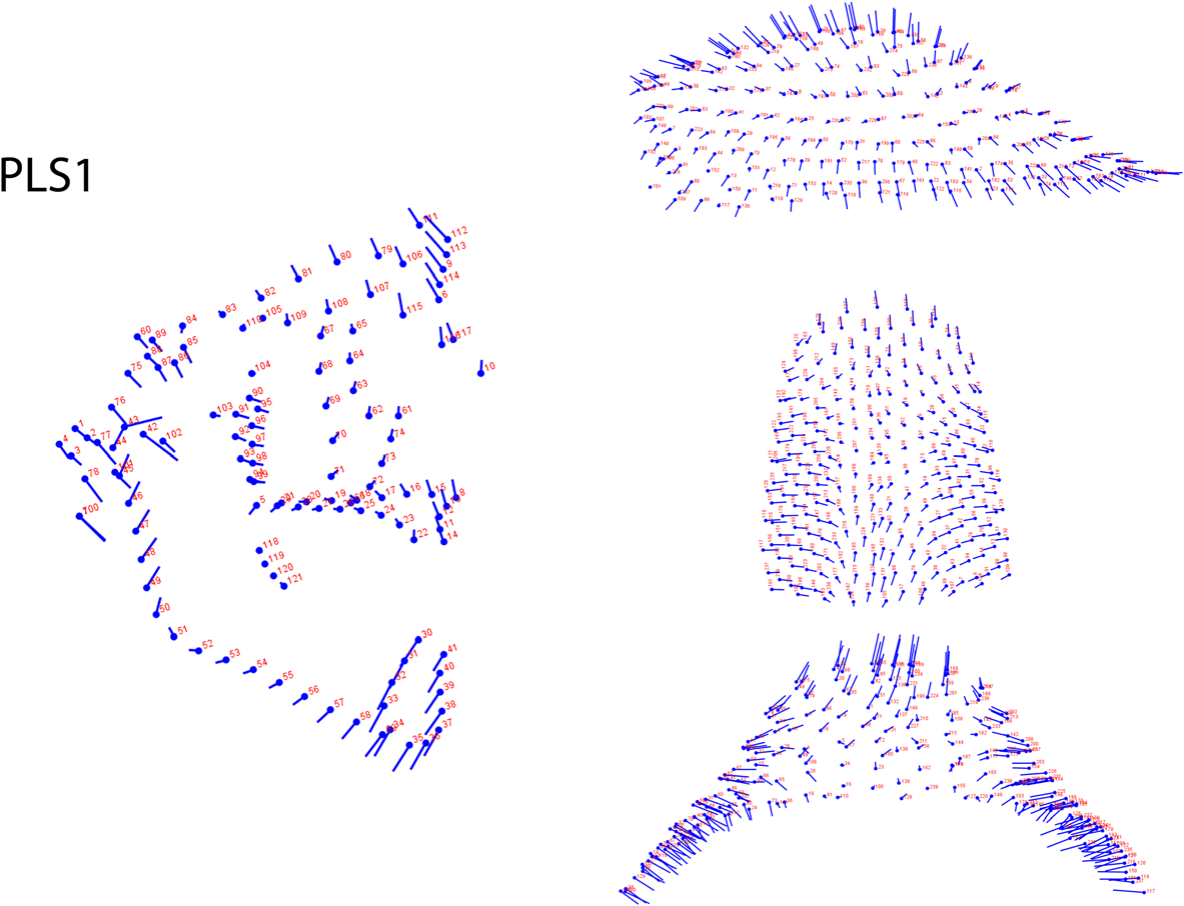
Graphic depiction of the PLS1: craniofacial divergence was related to palatal height and width

In our sample maxillary and craniofacial shape covaried significantly (RV coefficient: 0.1834). PLS1 represented the 61.7% of the whole covariation and joined the divergence of the craniofacial complex to the palatal height and width. The craniofacial shape changes were especially evident in the mandibular ramus, in the condylar and symphyseal mandibular regions. PLS1 demonstrated that in Class III subjects increments of angle divergence are related to a narrow and high palate (Fig. 7).

The leftover values were smaller and statistically insignificant.

## Discussion

This investigation evaluated the palatal and craniofacial morphological covariation in Class III growing patients by means of GMM.

In literature, Parcha et al. [14] studied this kind of shape covariation in a general orthodontic population, while Paoloni et al. [15] used the same methodology for a Class II malocclusion mixed dentition orthodontic population. To our knowledge, this is the first attempt to analyse the covariation between palatal and craniofacial shape in a group of growing subjects with Class III malocclusion.

Treatment outcomes for subjects with Class III malocclusion are dependent on multiple factors including growth characteristics, facial morphology, environmental factors, direction and magnitude of corrective forces, treatment timing and duration, and patient’s compliance [4–6]. While several studies [7–10] have been useful to understand Class III growth patterns, Class III craniofacial skeletal pattern needed more research, as it is a complex biological nonlinear system in which one component’s action changes the context for other components [25].

In fact, the variations in transverse, vertical, anteroposterior skeletal factors, and palatal morphology are complex and are related in various directions [16].

The first attempt of three-dimensional evaluation of mandibular changes in Class III malocclusion subjects was made by Singh et al. [26] on lateral cephalograms. The aim of their study was to apply the finite-element morphometry (FEM) to human mandibular configurations and determine local size- and shape-change differences in subjects with normal and Class III malocclusion between 5 to 11 years. FEM analysis revealed that the combination of a longer mandibular corpus and shorter ramus, associated with acute mandibular and symphyseal angles, distinguished a Class III mandible from a normal one.

Then, the thin-plate spline analysis (TPS) was used to evaluate the mandibular deformations in Class III subjects (adults and children) when compared to normal occlusion subjects [11]. The study showed in the Class III group a longer mandibular body and a narrower ramus, allied with a larger mandibular angle, combined to a longer mandibular total length with upward and forward extension of the ascending ramus and forward and downward extension of the mandibular symphysis [11].

Bui et al. [27] used a cluster analysis and a principal component analysis based on cephalometric variables to evaluate the most significant changes of the craniofacial complex in Class III malocclusion adults (mean age 19.10 years). Three PCs were selected: the first principal component consisted of sagittal parameters; the second principal component was significant for vertical measurements and for lower incisor position; the third principal component consists of variables related in both anteroposterior and vertical dimensions.

The studies described above, however, assessed only the craniofacial deformations, in particular the mandibular ones, without correlation to the palatal changes and they collected samples of Class III malocclusion adult patients.

Recently, Ahn et al. [16] used the SEM analysis, applied to CBCT and maxillary study models, to study the relationship between the morphology of the palate and the facial skeletal patterns in Class III malocclusion adult patients (mean age 22.12 years). The Authors observed that the palatal shape was narrow, deep, and long, or was wide, shallow, and short, depending on the transverse facial skeletal pattern. In contrast, the anteroposterior latent variable had a low influence on the principal component, in that the variation of the palatal morphology: even if the posterior facial height is long, its influence on palatal shape variation would not be significant.

On the contrary, our study focused on growing subjects with Class III malocclusion and used the two-block PLS method to evaluate the covariance between palatal and craniofacial components.

As suggested by Parcha et al. [14], subjects with unilateral crossbite and lateral mandibular shift were excluded because of their potential impact on palatal shape and asymmetry, such as patients with sucking habits or mouth-breathing pattern. The influence of the habits on craniofacial growth has been widely debated since in growing subjects they could influence the development of different palatal and craniofacial morphologies due to muscular and postural alterations [28].

In according to Ahn et al. [16], in our study palatal morphological changes occurred in all the three space dimensions (Fig. 3): a wide palate was related to a shallow palatal shape, while a narrow palate was associated with a high palatal vault. As for the morphology of the craniofacial complex (Fig. 5), the most significant morphological variability referred to the vertical and not to the sagittal plane. The analysis of the pattern of covariation demonstrated a statistically significant relation between the divergence of the craniofacial complex and the shape of the palate.

To our knowledge, in literature only two studies evaluated the covariation between the morphology of the palate and the facial skeletal patterns using different sample of malocclusion. Parcha et al. [14], analysing the palatal morphology and its relationship to skeletal pattern in a general orthodontic population, underlined that high and narrow palatal vaults were principally associated to a hyperdivergent skeletal pattern while shallow and wide palates to a hypodivergent one. Paoloni et al. [15] evaluated a group of Class II malocclusion growing patients, showing that the tendency to develop a transverse deficit of the maxilla was more easily recognizable in Class II subjects with high angle mandibular pattern. Despite the different analysed subjects, both the two authors found covariation results that were similar to the ones found in our study. We confirmed that beyond the kind of sagittal malocclusion there is a strong correlation between the maxillary morphology and the vertical facial skeletal pattern.

The clinician should be aware of the close correlation between the transverse and vertical dimension and should carefully analyse both discrepancies in the diagnostic process of Class III malocclusion. Since the maxillary skeletal base widths and the maxillary intermolar widths were significantly smaller in this malocclusion [10], these patients may more often require orthopaedic maxillary expansion [29–31]. Moreover, among the predictive variables of treatment stability, gonial angle and vertical growth have been included [32, 33]. An unfavourable prognostic pattern of Class III malocclusion has been identified by narrow maxilla, obtuse gonial angle, increase vertical skeletal pattern [32, 33]. Considering these conditions, the control of the vertical dimension in Class III malocclusion growing patients may be advisable.

### Limitations

Since the records were obtained from Class III individuals who chose to seek treatment and subjects who present for correction of their malocclusion, they may represent a more severe phenotype than occurs within the normal population. Moreover the study included only Caucasians so these results will not apply to other ethnic groups. As recommended by Parcha et al. [14], the palatal vault was assessed up to the gingival margin as described by previous studies in order to eliminate the influence of dental inclination and position in the alveolar bone.

Although we accomplished a comprehensive three-dimensional description of palatal shape, the use of two-dimensional cephalometric radiographs, which exclude the transverse dimension of the craniofacial complex, is a noteworthy limitation.

## Conclusions

Class III malocclusion growing subjects presented a statistically significant covariation between palatal and craniofacial morphology: in Class III subjects increments of angle divergence are related to narrow and high palates.

## Data Availability

The datasets used and/or analysed during the current study are available from the corresponding author on reasonable request.

## Abbrevations

GMM: Geometric morphometric analysis
SEM: Structural equation modelling
TPS: Thin-plate spline
PCA: Principal component analysis
PLS: Partial least squares
FEM: Finite-element morphometry
